# Pyorrhœa Alveolaris

**Published:** 1888-01

**Authors:** A. W. Harlan

**Affiliations:** Chicago, Ill.


### Pyorrhœa Alveolaris.

BY A. W. HARLAN, M.D., D.D.S., CHICAGO, ILL.

[Read before the Ohio State Dental Society, Springfield, Ohio, October, 1887.]

On short notice I have consented to address you to-day on the subject announced, and my only object in doing so is to keep alive the interest in the proceedings of this society.

From reading many reports of cases treated and the manner of handling the subject by numerous writers during the past few years, many, no doubt, have very vague ideas concerning the causes of loosening of the teeth. First it may be stated that the loosening of teeth brought about by deposits of salivary calculus around their necks seldom, if ever, is accompanied by exuding pus, which does not cease flowing immediately after the removal of the deposits. Second, in all cases of the so-called pyorrhœa alveolaris we have then to deal with conditions not existent as a result of salivary deposits. Third, alveolar pyorrhœa strictly defined is simply the flow of pus from between the gums or alveoli and the roots of teeth. Concomitant with this flow of pus we may have deposits on the roots, as little granules, sheets or rings around the teeth, caries and necrosis of the alveolar process and more or less thickening, and tumefaction of the gum tissue, with a detachment of the peridental membrane more or less complete, even to or around the apices of roots. Many cases present pockets leading from the gingival margin irregular in outline, which, when accidentally closed by particles of food or mucus or from any cause, may quickly set up an acute inflammation, so painful as to cause the loss of the tooth by extraction or hopeless loosening. The gums recede from the necks of the teeth after the loss of the alveolar process, and from impending operations, as well as from the use of escharotics, and other powerful medicaments. In many cases, however, no reunion is observable. The lower incisors are freer from true alveolar pyorrhœa than all other teeth in the mouth.

I will not rehearse the various theories concerning the etiology of pyorrhœa alveolaris or Riggs' disease as it is called, for you must be familiar with most of them ; still it may not be uninteresting to note that two papers have been presented to the Odontological Society of Great Britain recently by Mr. F. N. Pedley and Dr. E. Magitot ; the former after reviewing the prominent theories, says in conclusion that "The weight of evidence tends to place pyorrhœa alveolaris in the category of bone diseases. The exposed portion of the alveolar margin and its intimate relation with organs of such feeble vascularity as the teeth, go far to explain why it is this portion of the alveolus that is first affected, and also the usual arrest by the removal of the teeth. Dr. Magitot calls it "Symptomatic Alveolar Arthritis."

"Having referred to the history of, and the literature on the disease, the writer says these lesions consist, in fact, of a derangement of an inflammatory nature, affecting at the same time the tissue which has been called the *alveolo-dental periosteum*, and the bony covering which is subjacent to it, viz., the cement. The term osteo-periostitis was thus justified by the simultaneous lesion of a periosteal and osseous layer. The expression conformed, moreover, to the rule adopted in France on the subject of medical nosology, which gives to every disease a name corresponding to the anatomical lesions which characterize it.

Be that as it may, this last description does not appear to have satisfied all authors, for several of them have gone back to the term *expulsive gingivitis*, which is evidently incorrect, since the gum is never primarily attacked. Others, again, have taken up the term *alveolar pyorrhœa*, which term seems to prevail amongst English practitioners, and particularly with our colleague, Mr. F. Newland Pedley; whilst others, Dr. David, of Paris, for example, propose to call it Fauchard's disease (*Maladie de Fauchard*), after the name of the author, who was one of the first to mention it as described. Following the same idea it is called in England and America Riggs' disease.

If this tendency to multiply without any definite rule the names of the same disease be persevered in much longer, one calling it from one of its symptoms *pyorrhœa* or *suppuration*, others giving to it the name of an author who has described it with more or less exactitude, extreme confusion will be the result.

Moreover, amongst this diversity of titles the rule referred to above, of invariably taking as the basis of nomenclature the pathological anatomy of disease, is completely forgotten.

Thus, while regretting to add in our turn a new name to a nomenclature already so long, we feel ourselves, nevertheless, obliged to substitute once more for the designation hitherto employed a title which, faithfully conforming to the precise rules indicated, will establish the nature of the disease with all the exactitude desirable. This title is that of *Symptomatic Alveolar Arthritis*.

The justification of this choice is very simple. The recent researches made in France, particularly those by Mons. Malossez, have clearly established the fact that that which we have described as *periosteum* around the roots of the teeth ought to be considered, not as a membrane, distinct and capable of being dissected off, like the osseous periosteum, but rather as a true ligament.

This manner of looking at it, already indicated by Kolliker in Germany, and by Ranvier in France, in his course of lectures at the College of France, is generally adopted in our country. We, ourselves, are in complete harmony with it, thus accepting the necessity of modifying in our own studies the interpretation which we had, until now, assigned to this part of the dental organ.

As we have already indicated in our previous researches, the causes of this disease should be looked for, not in the local conditions of the mouth or gums, but in certain conditions of the general health. The disease ordinarily attacks a single tooth; occasionally several teeth may become involved, but in the latter case the affected teeth are not necessarily contiguous, they may occupy different positions in the mouth at a distance from each other. Toirac and M. Oudet believe they have observed that the inferior incisors were more particularly the seat. We have not ourselves recognized this peculiarity, which appears to us to belong more especially to gingivitis, which may in certain cases be confounded with symptomatic alveolor arthritis.

The teeth most frequently affected are in order of sequence: firstly, the molars, then the inferior incisors, the bicuspids, the superior incisors, and lastly the canines. We have never observed this disease affecting simultaneously the whole of the teeth. At one time it is situated on one or two inferior or superior incisors, at another the incisors are spared, and one or more molars are affected, ordinarily two or three on different sides of the mouth. Sometimes the disease attacks only a single root of these latter, or it may be only a single side of a root, a circumstance which preserves to the root a certain degree of solidity for a considerable time. The teeth affected with symptomatic alveolar arthritis do not generally present any preceding or accompanying alteration. Caries, for instance, has no connection with this disease, and if this complication is present it is simply an accidental occurrence. It is, indeed, worthy of remark that the local conditions which accompany the development of symptomatic alveolar arthritis appear to be just the opposite to those which accompany the production of caries; the buccal saliva is in fact rather alkaline than acid, an accumulation of tartar more or less abundant is observed in the places where it is usually met with. One might be tempted on the first glance to attribute to this deposit a part more or less active in the etiology of this disease, but it is not so. The deposit of tartar is a secondary matter, and in all cases, its formation being generally uniform and continuous in the same region, it cannot be cited in the production of an isolated and local affection.

Gouty and rheumatic subjects often present this kind of arthritis, as also do individuals attacked with anæmia caused by long illness; but there are no general lesions which exercise a graver influence on the production of this disease than those of albuminuria and, above all, diabetes. As regards the first we are dealing here, as is well understood, not with symptomatic albuminuria, but with what is properly called Bright's disease.

We are in fact confronted with a septic and contagious disease, of which the essential symptoms are an abundant alveolar suppuration, loosening and displacement of the teeth, recession of the gums—in a word, all the conditions suitable to make of this lesion a spot favorable for the action of septic agents.

These agents undoubtedly exist in this disease. The microscopic preparations of pus proceeding from alveolar diseases have betrayed the existence of a large number of organisms of very diverse forms, amongst which it is, if the truth be spoken, still difficult to distinguish the variety which ought to be regarded as the essential and exclusive morbid agent of this disease. Further researches, we are convinced, will enable us to realize the culture of the pathogenic agent peculiar to symptomatic alveolar arthritis.

Some interesting experiments, undertaken by MM. Malassez and Galippe, for this purpose have already put beyond doubt the parasitic nature of this disease, and it is also confirmed by the frequent propagation of the disease from one alveolus to another in the same mouth, as well as by contagion from one individual to another, as observed by different authors and ourselves. All that we now wish to insist upon is the value of antiseptics in the treatment of this disease. With this view we would recommend such applications as alcohol, carbolic acid, perchloride of mercury, etc. For this purpose we have selected the following formulæ:

| | | | |
|---|---|---|---|
| No. 1. ℞ | Hydr. perchlor. | - - | 50 c.g. |
| | Aq, Distil. | - - - | 1,000 grms. |
| No. 2. ℞ | Acid carbolic crystal | - } | —— |
| | Either | - - - - } | aa 5 grm's |
| | Alcohol | - - - - | 10 " |
| No. 3. ℞ | Acid borac. | - - - | 10 " |
| | Aq. Distil. | - - - | 500 " |
| | Alcohol | - - - - | 50 " |

To these formulæ we could have very well added others by employing other antiseptics, such as the permanganates, salicylates, salicylic acid, etc. It will be necessary besides to have recourses to the employment of medicines which will modify the the local condition, as well as to attend to the treatment of the general health, paying due regard to the diathesis which influence the disease.

Thus the treatment of symptomatic alveolar arthritis can be summed up in the three following paragraphs:

1st. To render anticeptic the alveolus, the seat of the arthritis.

2nd. To modify the condition of the affected tissues by the application of astringents and caustics.

3rd. To treat the general conditions or diatheses which govern the local manifestations.

These are his conclusions: 1st. The affection characterized by alveolar suppuration, by the loosening and falling of the teeth, ought to be considered as a true symptomatic alveolar arthritis, septic and contagious, and which ought henceforth to remain known by that name in surgical nosography.

2nd. This affection is most often met with under the influence of certain general conditions and diatheses, and as secondary to eruptive fevers, etc., of which it may form either a complication or sequella.

3rd. The therapeutics of symptomatic alveolar arthritis ought to consist essentially in the employment of antiseptics, of local alternatives, astringent, or caustics, without prejudice to the treatment of the general conditions which are the determining causes."

These extracts are presented without comment on account of the want of time to consider them properly. In face of the recent work of Dr. Black on the peridental membrane the view that it is a ligament is simply ridiculous, even such a high authority as Dr. Magitot by giving the weight of his name to the unsupported theory of Malassez, that this membrane is ligamentous makes it highly improbable that this new name, based on a histological myth will be adopted. Were it worth while we could show from comparative embryology anatomy and histology that the position was untenable. To step aside for a moment and let the theories take care of themselves, you must all realize what a dreadful malady we have to deal with. Caries of the teeth is not nearly so destructive as loosening of the teeth on account of the ease of arresting it. For genuine discomfort, and general disgust with himself a real case of badly loosened teeth, with pus oozing into the mouth and a foul smell proceeding therefrom, is to a refined and highly organized person about as bad as any other local malady you can think of. These cases are seen almost every day in various stages, and we must do something to help the sufferers. Too little attention is paid to such cases to be of much benefit to the patient. Dentists, as a rule, are not sufficiently bold in operating and they are weak therapeutically. The instruments for operating are generally too clumsy and they are unskillfuly handled; too much is attempted at one sitting. It is far better that two or three teeth should be freed from seminal deposits and the bone excised or scraped and the medicines brought in contact with the surfaces operated upon, rather than fail to accomplish anything by operating on a dozen teeth in the same length of time if there be necessity for it; success will not follow any operation unless conscientiously performed.

The caustics and escharotics and powerful astringents are not indicated *in* the pockets, but when used they should be applied to the edges or outside on the surfaces of the gums. Perfect cleanliness of instruments, removal of all debris from the mouth and around or between the teeth, the use of washes sufficiently constituted so as to cause a free flow of saliva into the mouth, the regular medication of the diseased surfaces, not too frequent to destroy newly forming tissue, proper diet, exercise and open bowels are all essentials in the treatment of pyorrhœa alveolaris or so-called Rigg's disease.

